# Variation in wild pea (*Pisum sativum* subsp.* elatius*) seed dormancy and its relationship to the environment and seed coat traits

**DOI:** 10.7717/peerj.6263

**Published:** 2019-01-14

**Authors:** Iveta Hradilová, Martin Duchoslav, Jan Brus, Vilém Pechanec, Miroslav Hýbl, Pavel Kopecký, Lucie Smržová, Nikola Štefelová, Tadeáš Vaclávek, Michael Bariotakis, Jitka Machalová, Karel Hron, Stergios Pirintsos, Petr Smýkal

**Affiliations:** 1 Department of Botany, Palacký University Olomouc, Olomouc, Czech Republic; 2 Department of Geoinformatics, Palacký University Olomouc, Olomouc, Czech Republic; 3 The Centre of the Region Haná for Biotechnological and Agricultural Research, Crop Research Institute, Prague, Olomouc, Czech Republic; 4 Department of Mathematical Analysis and Applications of Mathematics, Palacký University Olomouc, Olomouc, Czech Republic; 5 Department of Biology and Botanical Garden, University of Crete, Heraklion, Greece

**Keywords:** Dormancy, Seed coat, Proanthocyanidins, Testa, Pea, Niche-modelling, Temperature oscillations, Germination, Legumes

## Abstract

**Background:**

Seed germination is one of the earliest key events in the plant life cycle. The timing of transition from seed to seedling is an important developmental stage determining the survival of individuals that influences the status of populations and species. Because of wide geographical distribution and occurrence in diverse habitats, wild pea (*Pisum sativum* subsp.* elatius*) offers an excellent model to study physical type of seed dormancy in an ecological context. This study addresses the gap in knowledge of association between the seed dormancy, seed properties and environmental factors, experimentally testing oscillating temperature as dormancy release clue.

**Methods:**

Seeds of 97 pea accessions were subjected to two germination treatments (oscillating temperatures of 25/15 °C and 35/15 °C) over 28 days. Germination pattern was described using B-spline coefficients that aggregate both final germination and germination speed. Relationships between germination pattern and environmental conditions at the site of origin (soil and bioclimatic variables extracted from WorldClim 2.0 and SoilGrids databases) were studied using principal component analysis, redundancy analysis and ecological niche modelling. Seeds were analyzed for the seed coat thickness, seed morphology, weight and content of proanthocyanidins (PA).

**Results:**

Seed total germination ranged from 0% to 100%. Cluster analysis of germination patterns of seeds under two temperature treatments differentiated the accessions into three groups: (1) non-dormant (28 accessions, mean germination of 92%), (2) dormant at both treatments (29 acc., 15%) and (3) responsive to increasing temperature range (41 acc., with germination change from 15 to 80%). Seed coat thickness differed between groups with dormant and responsive accessions having thicker testa (median 138 and 140 µm) than non-dormant ones (median 84 mm). The total PA content showed to be higher in the seed coat of dormant (mean 2.18 mg g^−1^) than those of non-dormant (mean 1.77 mg g^−1^) and responsive accessions (mean 1.87 mg g^−1^). Each soil and bioclimatic variable and also germination responsivity (representing synthetic variable characterizing germination pattern of seeds) was spatially clustered. However, only one environmental variable (BIO7, i.e., annual temperature range) was significantly related to germination responsivity. Non-dormant and responsive accessions covered almost whole range of BIO7 while dormant accessions are found in the environment with higher annual temperature, smaller temperature variation, seasonality and milder winter. Ecological niche modelling showed a more localized potential distribution of dormant group. Seed dormancy in the wild pea might be part of a bet-hedging mechanism for areas of the Mediterranean basin with more unpredictable water availability in an otherwise seasonal environment. This study provides the framework for analysis of environmental aspects of physical seed dormancy.

## Introduction

The transition from seed to seedling is one of the most important stages in the plant life cycle (Donohue et al., 2010). This transition is a complex process influenced by structural and physiological characteristics of the seed, as well as by genetic and environmental factors. This stage constitutes one of the most dramatic changes of the plant life cycle, involving the switch from a robust, quiescent state of the protected seeds to one of extreme vulnerability, the young seedling. Therefore it is essential that germination takes place at the appropriate time and place (Fenner & Thompson, 2005). Seed dormancy provides the mechanism blocking germination of intact viable seeds under conditions when the probability of seedlings survival and growth is low (Baskin & Baskin, 2004; Weitbrecht, Müller & Leubner-Metzger, 2011; Bewley et al., 2013; Long et al., 2015). A diverse range of dormancy mechanisms have evolved as response to the diversity of climates and habitats (Baskin & Baskin, 2004; Willis et al., 2014). Physical dormancy (PY) occurs in at least 18 angiosperm plant families including Fabaceae (Baskin & Baskin, 2014; Willis et al., 2014) and is caused by a layer of water-impermeable palisade cells in the seed coat (reviewed in Smýkal et al., 2014; Janská et al., 2018). Phylogeny based analysis of world-wide observations of Fabaceae identified the evolution towards non-dormant seeds in climates with long growing seasons such as the ones found in the tropics (Rubiode Casas et al., 2017), suggesting that the seed dormancy might be favored as the bet-hedging strategy in temporally variable environments (Venable, 2007; Rubiode Casas et al., 2017). In such circumstances, dormancy may prevent inopportune germination by delaying germination until the onset of the autumn-winter rainy season as found in Mediterranean region (Thompson, 2005). Association between seed responsiveness to temperature and the thermic characteristics of their habitat range has been reported (Fenner & Thompson, 2005; Bewley et al., 2013; Baskin & Baskin, 2014; Batlla & Benech-Arnold, 2015; Renzi, Chantre & Cantamutto, 2018; Thompson, 1970; Probert, 2000; Rosbakh & Poschlod, 2015). These studies have shown that temperature determines the sensing of the time of the year and depth of seed dormancy. Seeds of native plants in highly seasonal climates, such as the Mediterranean climate, experience both daily and seasonal fluctuations in temperature and moisture (Ader, 1965; Norman, Cocks & Galwey, 2002). Such temperature oscillations have been proposed to be one of the PY dormancy breaking mechanisms (Baskin & Baskin, 2014). The positive effect of temperature on seed dormancy release was confirmed experimentally in several legume species, including *Lupinus, Trifolium* and *Medicago* (Quinlivan, 1961; Quinlivan & Millington, 1962; Quinlivan, 1966).

In addition to temperature, the water availability is another important environmental factor, related to water potential of both the soil and seed, which also regulates dormancy (Bradford, 2002). Furthermore there are other soil factors that contribute to the complexity of water availability, these include physical (temperature, texture, gaseous environment, and seed burial depth), chemical (organic matter, pH, and nutrients) (Long et al., 2015) and biological soil (fungal and microbial activity) properties (Sperber et al., 2017).

Geographical gradients of environmental factors such as temperature and precipitation offer great opportunities for evaluating inter-population variability in plant traits and provide the possibility to analyze association between studied traits, environment and genetic constitution of the individual. Such studies have been conducted in species with sufficiently large geographical distribution and/or wide niche breath such as *Arabidopsis* (Exposito-Alonso et al., 2018; Tabas-Madrid et al., 2018; Frachon et al., 2018), *Medicago* (Yoder et al., 2014) or *Populus* (Fitzpatrick & Keller, 2015). With respect to seed dormancy, this was studied in *Arabidopsis* model (Vidigal et al., 2016; Burghardt et al., 2015; Donohue et al., 2010) and sea beet (*Beta vulgaris* subsp*. maritima*, Wagmann et al., 2012), but so far not on legume seeds with physical dormancy type.

Structural and compositional properties of the seed coat have been reported to affect the seed dormancy status (reviewed in Smýkal et al., 2014; Hradilová et al., 2017; Janská et al., 2018). Particularly polyphenols have been found in the seed coat, such as flavonoids, lignins and lignans and there is evidence that these influence both seed longevity and dormancy (Lepiniec et al., 2006). The relationship between proanthocyanidin (PA) content and dormancy was shown in *Arabidopsis* (Appelhagen et al., 2014), rapeseed (*Brassica napus*) and flax (*Linum usitatissimum*) mutants (Diederichsen & Jones-Flory, 2005; Zhang et al., 2006), faba bean (Kantar, Pilbeam & Hebblethwaite, 1996) and also pea (Hradilová et al., 2017). These PA insoluble polymers and other compounds (Cechová et al., 2017) result in the reinforcement of the seed coat as a barrier to water and oxygen permeation, mechanical damage and biotic and abiotic stresses (Pourcel et al., 2007). The association between the seed coat structure and composition in relation to dormancy and environmental factors across the species distribution range was not tested until now. Moreover, structural and compositional properties of the seed coat can be influenced by the intraspecific or even intra-individual variation in seed size (Mandák, 1997; Baskin & Baskin, 2014). In *Atriplex trianglularis*, thickness of seed coat increases with decreasing seed size (Khan & Ungar, 1985). Consequently, larger mechanical resistance of testa of smaller seeds in *Atriplex* provides a resistance to germination and might induce dormancy in a smaller seeds (Osmond, Björkman & Anderson, 1980). The seed size also relates to water requirements during imbibition, presumably due to surface-to-volume ratio (Wilson & Witkowski, 1998). Indeed, it has been hypothesized that level of dormancy and seed size are interrelated and have adaptive values (Venable & Brown, 1988; Rees, 1996).

Wild pea (*Pisum sativum* subsp. *elatius*), with its wide ranging native distribution, spanning from the western Mediterranean, through the southeastern Europe to the Middle East (Smýkal et al., 2017; Smýkal et al., 2018a) offers an excellent model to study legume seed physical dormancy in an ecological context. Given the limited knowledge on regulation of legume seed dormancy (reviewed in Smýkal et al., 2017) this study addresses the gap in knowledge on association between the seed dormancy, seed properties and environmental factors. The present study had three objectives: (1) To describe germination response of pea seeds to various experimental temperature treatments. (2) To determine which ecological factors (climate, soil conditions) may act as adaptation drivers of seed dormancy level in wild pea. (3) To test if there is an association between seed dormancy level variation, seed coat anatomy and proanthocyanidin content.

## Materials & Methods

### Plant material

**Figure 1:**
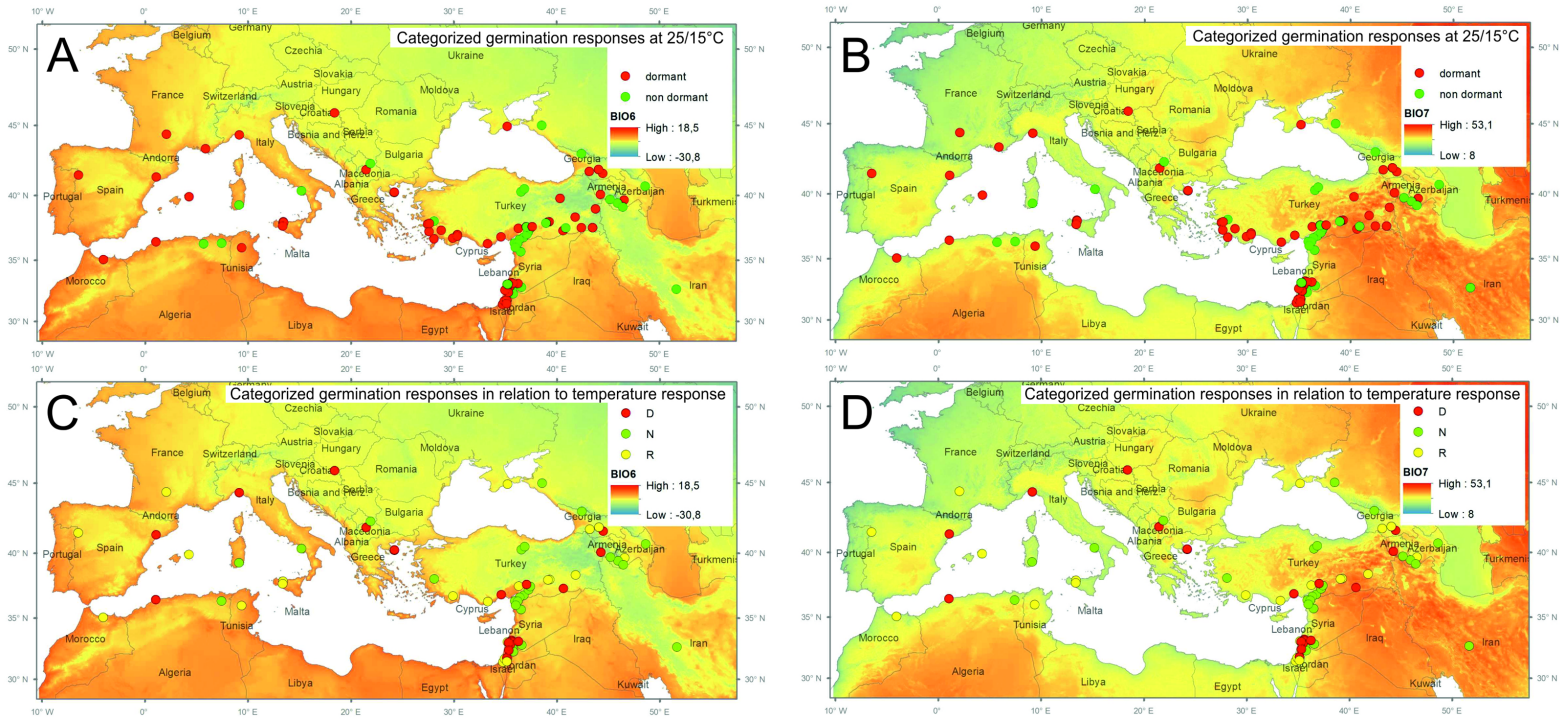
Spatial distribution of tested samples and their categorized germination pattern in relationship to selected bioclimatic variables. Categorized germination patterns of accessions tested at alternate temperature regimes 25/15 °C (A, B) and 35/15 °C (C, D) over smoothed bioclimatic variables BIO6 (Min Temperature of Coldest Month) and BIO7 (Temperature Annual Range) extracted from WorldClim 2.0 database. (D, dormant, N, non-dormant, R, responsive accessions. See Table S2 for details).

The investigated samples originated from various genebanks and contained 97 wild pea (*Pisum sativum* subsp. *elatius* (M. Bieb.) Asch. & Graebn) accessions. These span from the western Mediterranean, through the southeastern Europe to the Middle East (Table S1, Fig. 1). This material was selected based on passport data including GPS information and assessed for genetic diversity relationship (Smýkal et al., 2017; Smýkal et al., 2018a; Trněný et al., 2018). Plants were grown in 5 litre pots with peat-sand (90:10) substrate mix (Florcom Profi, BB Com Ltd. CZ), in glasshouse conditions (January–May 2016 and 2017) day/night temperature within the ranges 35–20/15–12 °C, and a natural photoperiod increasing from 6 to 14 h without supplementary light (UP campus, Olomouc, CZ). Cultivated and wild peas are largely self-pollinating (Smýkal et al., 2018a) and as samples were of single seed descent they are expected to be genetically uniform. After harvest, mature seeds were cleaned from pods, dried at room temperature and packed in paper bags. The germination testing was performed within the month from harvest.

### Germination testing

To assess the dormancy status, seed imbibition and germination was assessed (Baskin & Baskin, 2014). Based on temperature data from soil sensors monitored at several sites with wild pea populations (Smýkal et al., 2017; Von Wettberg et al., 2018) we tested the effect of two oscillating temperature regimes on dormancy level: (i) day/night temperatures of 35/15°C and (ii) 25/15 °C, both at 14h/10 h periodicity and in the dark. Intact seeds were placed on water saturated filter papers (Whatman Grade 1, Sigma CZ) in 90 mm Petri dishes (P-Lab, CZ) in temperature controlled chambers (Laboratory Incubator ST4, BioTech CZ). For each treatment, 25 seeds per accession with two replicas were incubated. To prevent fungal growth the fungicide (Maxim XL 035 FS; containing metalaxyl 10 g and fludioxonil 25 g) was applied in amount of 1 ml l^−1^ of tap water. Initial comparison of control and fungicide containing samples did not show any significant difference. Seeds were monitored at 24 h intervals for a total of 28 days (the longest time the seeds remained fungus-free in the 35/15 °C treatment) and water was added as needed usually every 3rd day. The plates were randomly rearranged during the scoring.

### Germination data analysis

The number of imbibing seeds (i.e., swollen enlarged seeds upon the uptake of water) and also germinating seeds (i.e., radicle protrusion was the criterion for germination) was scored daily for the period of 28 days for each accession. Since we are primarily interested in study of physical dormancy (PY) executed by seed testa barrier of water entry (Smýkal et al., 2014) we have used the number of imbibed seeds as a measure of germination in all further calculations (thus our data contain both imbibed and fully germinated seeds, which can be separated by several days from each other, notably all imbibed seeds have germinated in course of experiment).

In order to capture the complexity of germination we have used a *spline function* (function defined piecewise by polynomials) fitted to the data (Ramsay & Silverman, 2005; Van den Boogaart, Egozcue & Pawlowsky-Glahn, 2014). This treats the original germination data (daily counts of germinated seeds) as discrete realizations of an asymptotically continuous process, i.e., for each sample the germination can be represented by a non-decreasing function called the *absolute germination distribution function* (AGDF) in the following text. The term *absolute* is used to distinguish AGDF from standard cumulative distribution function; unlike the latter one, AGDF deals with absolute values of cumulative germination. This function reflects well specific patterns related to germination of a given accession including final germination proportion and germination speed. In addition, we considered the area under AGDF, computed using the Riemann integral. From its construction, increasing value of this coefficient indicates both higher germination proportion and/or germination speed. For its numerical computation we used numerical integration, composed trapezoidal rule (Burden & Faires, 2011). AGDFs were obtained by approximation of the original germination data by smoothing with spline functions (De Boor, 1978). We used a generalized functional (Machalová, 2002) with the smoothing spline of degree *k* = 4 (degree of the respective basis polynomials used for construction of the splines) and the first derivative in *L*_2_ norm in Matlab (https://www.mathworks.com/) and further processed using R (R Development Core Team, 2018). Each spline is characterized by a set of real numbers, called B-spline coefficients. They represent coefficients of a linear combination of basis functions (so called B-splines) which are the same for all samples and each of them covers part of the domain of the germination function. Accordingly, a value of each specific coefficient indicates local behavior of the spline (and, consequently, of the original germination data). Moreover, B-spline coefficients can be also further tuned, like here to achieve better smoothing properties. B-spline coefficients for each accession (Table S2) were subjected to cluster analysis using UPGMA and Euclidean distance metric (Legendre & Legendre, 2012) separately for each temperature treatment, while areas under the curve were used in the case of combined temperature treatments dataset. Therefore, concrete values of B-spline coefficients were not important for interpretation purposes here, however, they were simply utilized for the above multivariate analyses. Cluster analyses were performed in the software PAST 3.21 (Hammer, Harper & Ryan, 2001).

### Environmental variables

Environmental variables (climate: http://worldclim.org/version2; soil: https://soilgrids.org) were obtained using the ArcGIS Pro software (https://pro.arcgis.com). Species occurrence databases have often certain degree of uncertainty due to different spatial accuracy (Stine & Hunsaker, 2001; Murphey et al., 2004; Meyer, Weigelt & Kreft, 2016). In order to minimise the error caused by coordinates we developed a geoprocessing model to automate the calculation of mean values from 5 km buffer around the collection sites. Using this approach, we smoothed the uncertainty caused by imprecise localisation. Data were extracted using geoprocessing model from GeoTIFF rasters in the WGS-84 coordinate system (EPSG: 4326) with a spatial resolution of 30 arc-seconds (∼1 km). The bioclimatic variables (BIO1–BIO19), are derived from the monthly temperature and rainfall values (Fick & Hijmans, 2017). These represent annual trends (e.g., mean annual temperature BIO1, annual precipitation BIO12), seasonality (e.g., annual range in temperature and precipitation BIO4 and BIO15) and extreme or limiting environmental factors (e.g., the temperature of the coldest and warmest month BIO5 and BIO6, and amount of precipitation in the wet and dry quarters BIO16 and BIO17). Soil data were extracted from SoilGrids collection of updatable soil property and class maps of the world (Hengl et al., 2017). We used values described in Table S1 from 5 cm depth and resolution 1 km (same as WorldClim). For details, including basic descriptive statistics of mean tendency and variation of each environmental variable and legend see Table S1.

### Testing of relationships among germination pattern, geography and environment

Matrix of environmental variables was firstly checked for the presence of the multicollinearity. Because of strong covariation between several environmental variables (pairwise Pearson *r* ≥ |0.90|), eight soil and nine climatic variables were removed from the dataset and all remaining analyses (except for niche analysis) were done with reduced matrix of environmental variables containing 10 climatic and nine soil variables (Table S1). To assess whether there is spatial correlation present in the data, Moran’s I spatial correlation statistics (Legendre & Legendre, 2012) was calculated for each environmental variable in the reduced matrix using the software PASSaGE v. 2.0 (Rosenberg & Anderson, 2011). Ten distance classes with unequal widths were created and Moran’s I was calculated for each distance class with largest class excluded. To test the global significance of each spatial correlogram, we check whether the correlogram contains at least one correlation statistics that is significant at the Bonferroni-corrected significance level (Legendre & Legendre, 2012).

The matrix of B-spline coefficients describing pattern of germination of the studied accessions in two temperature treatments (35/15 °C and 25/15 °C), considered as response variables, was analysed by Principal Component Analysis (PCA; Legendre & Legendre, 2012) to find the main gradients within the germination data-set. No standardization was applied on the matrix of B-spline coefficients prior to PCA. Moran’s I spatial correlation statistics was used with the same settings as for environmental variables to test the presence of spatial structuring of accessions scores on the first two resulting ordination axes (PC1, PC2). Set of climatic and soil variables and geographic coordinates (latitude, longitude) were used as supplementary variables and correlated with the first two principal components. To control of possible spatial autocorrelation between each environmental variable and principal components representing germination pattern, we used both standard and modified version of the *t*-test to assess the significance of correlation between two spatial processes. Modified version of the *t*-test (Dutilleul et al., 1993) was performed in PASSaGE v. 2.0 and PCA was performed using Canoco 5.10 (Ter Braak & Šmilauer, 2012).

Constrained ordination (redundancy analysis, RDA; Šmilauer & Lepš, 2014) was used to partition the variation in the matrix of B-spline coefficients explained by space (spatial position of sampling sites) and primary predictors (environmental variables), using PCNM method (Borcard & Legendre, 2002) without and with forward selection also for primary predictors. To identify spatial explanatory variables, we used principal coordinates of neighbouring matrices (PCNM; Borcard & Legendre, 2002), resulting in the PCNM variables. Only those PCNM variables with an adjusted P lower than 0.05 during forward selection procedure were selected to enter RDA. Subsequently, pure and shared effects of environment (groups of soil and climatic variables were tested separately) and spatial variables (PCNM) on the matrix of B-spline coefficients were analysed using RDA. We used a specific partitioning algorithm that provides unbiased estimates of explained variation in RDA (Peres-Neto et al., 2006). Here, in the first step, all environmental variables (either soil or climatic) from reduced data-set entered analysis as primary predictors. In the second step, a stepwise procedure using forward selection was done resulting in the subset of environmental predictors, best explaining the matrix of B-spline coefficients. P values were corrected using the False Discovery Rate adjustment (Benjamini & Hochberg, 1995). The significance of pure and shared (marginal) effects of the explanatory variables was tested by a Monte Carlo permutation test with 999 permutations. Analyses were performed in Canoco 5.10 (Ter Braak & Šmilauer, 2012; Šmilauer & Lepš, 2014) and the library *vegan* (Oksanen et al., 2018) in R.

### Niche analysis

Using the geographic locations of the studied accessions and their categorized germination response based on 35/15 °C treatment, we constructed ecological niche models for each of the germination response categories. As the modelling approach, Maxent (version 3.4.1, Phillips, Dudík & Schapire, 2017), a maximum-entropy based machine learning method was used. Maxent was shown to perform better than other methods when samples sizes are small (Elith et al., 2006; Hernandez et al., 2006) and it estimates the potential niche instead of the realized distribution of the modelled entity (Phillips, Anderson & Schapire, 2006). A buffer zone of 300 km was defined as the background area for each group. As environmental predictors, 19 bioclimatic variables, extracted from WorldClim (Fick & Hijmans, 2017) at a resolution of 2.5 arc minutes, were used. The equivalency tests (Warren, Glor & Turelli, 2008) were performed with software ENMTools (version 1.4.4 Warren, Glor & Turelli, 2010). Two overlap indices were employed in these tests, Schoener’s statistic, D (Schoener, 1968) and modified Hellinger distance, I (Warren, Glor & Turelli, 2008). Data manipulation and map creation were performed in the R with the packages sp (Pebesma & Bivand, 2005), raster (Hijmans, 2017) and dismo (Hijmans et al., 2017).

### Quantification of soluble and insoluble proanthocyanidins in the seed coat

Seeds of 86 accessions (Table S3) were analyzed for soluble proanthocyanidins (PAs, in acetone and 4-dimethylaminocinnamaldehyde [DMACA] reagent) and insoluble PAs (in butanol-HCl) using the method described by Pang et al. (2008). Briefly, separated seed coats were freeze dried and 20 mg was homogenized and extracted with 10 ml of acetone-acetic acid-water and 1 h sonication, followed by 5 min centrifugation at 10,000 rpm. To the 0.5 ml of extract, the 1.5 ml of 0.1% DMACA (Sigma-Aldrich, Karlin, Czech Republic) dissolved in HCl-ethanol-water solution, was added. The reaction was measured on spectrophotometer after 10 and 25 min at 640 nm using proanthocyanidin A (Sigma-Aldrich, Karlin, Czech Republic) as standard to calculate the amount of soluble PAs. The pellet after extraction of soluble PA was freeze dried and extracted with 5 ml of butanol-HCl (*x* − *yv*∕*v*), sonicated for 1 h, centrifuged (5 min/10,000 rpm) and measured at 550 nm. Thereafter the extract was boiled in a water bath for another 1 h, cooled down and the absorbance measured again at 550 nm using proanthocyanidin A as standard to calculate the amount of insoluble PAs. The total content of PAs among dormancy categories was compared by ANOVA. Ratio soluble/insoluble PA among dormancy categories was compared by two AN(C)OVA models, one with and the second without using total PA as a covariate. Analyses were performed in NCSS 9 (NCSS, Kaysville, UT, USA).

### Seed size and seed coat thickness measurements

Ten individual seeds per accession of all together 86 accessions (Table S3) were photographed using an Olympus SZ61 stereo microscope (Olympus Corp., Tokyo, Japan) equipped with an Olympus E-410 digital camera (Olympus Imaging Corp., Tokyo, Japan). The photographs were processed by QuickPHOTO MICRO 3.0, supplemented by the Deep Focus 3.3 module (PROMICRA s.r.o, Prague, Czech Republic). Image analysis of seeds was done by SmartGrain (Tanabata et al., 2012) recording seed area (AS), perimeter length (PL), width (W), length (L), length-to-width ratio (LWR), circularity (CS) and distance between intersection of length and width and centre of gravity (DS). The weight of twenty five seeds per accession with three replicas was recorded and converted to hundred seeds weight (HSW). Seed coat thickness of the stripped seed coat from five seeds per accession was measured in triplicate using a precision micrometer (0–25 mm, precision 0.01 mm, Hommel Herculer, Germany) and sectioned on cryo-microtome according to Hradilová et al. (2017). For the analysis of dormancy with respect to morphological parameters of seeds we used ordinal logistic regression (Kleinbaum & Klein, 2010) implemented in R package MASS (Venables & Ripley, 2002). Regression parameters were estimated using the maximum likelihood principle. The relative quality of specific models was assessed by Akaike information criterion (Akaike, 1974).

## Results

### Effect of temperature on pea seed germination

Ninety seven accessions were tested over a period of 28 days and the final percentage of germinated seeds ranged from 0% to 100% (Table S2). Cluster analysis of B-spline coefficients representing germination responses divided the accessions into two groups in the 25/15 °C temperature treatment (Fig. S1A). One cluster held 29 non-dormant (N) accessions with a mean germination of 92% and the second cluster had 68 dormant (D) accessions with a mean germination of 15% (Table S2). The non-dormant group was further divided into those that germinated quickly (N1, 13 accessions, with over 80% germinated at 10 days and 100% at 28 days) and more slowly (N2, 16 accessions germinating between 50 to 80% at 10 days and up to 80% at 28 days) (Table S2). The group of dormant accessions was further divided into D1 sub-group of 14 accessions with a mean germination of 31%, D2 subgroup of 47 with a mean germination of 6% and D3 subgroup of 7 accessions with a mean germination of 54%. In D2 subgroup there were 16 accessions which did not imbibed and germinated at all during 28 days of testing.

In the 35/15 °C temperature treatment, cluster analysis of B-spline coefficients resulted in two main clusters: one with 36 non-dormant (N) accessions with a mean germination of 95% and second with 61 dormant (D) accessions with a mean germination of 28%. The group of non-dormant accessions contained 29 accessions classified as non-dormant already at 25/15 °C, plus additional seven accessions (Fig. S1B).

**Figure 2:**
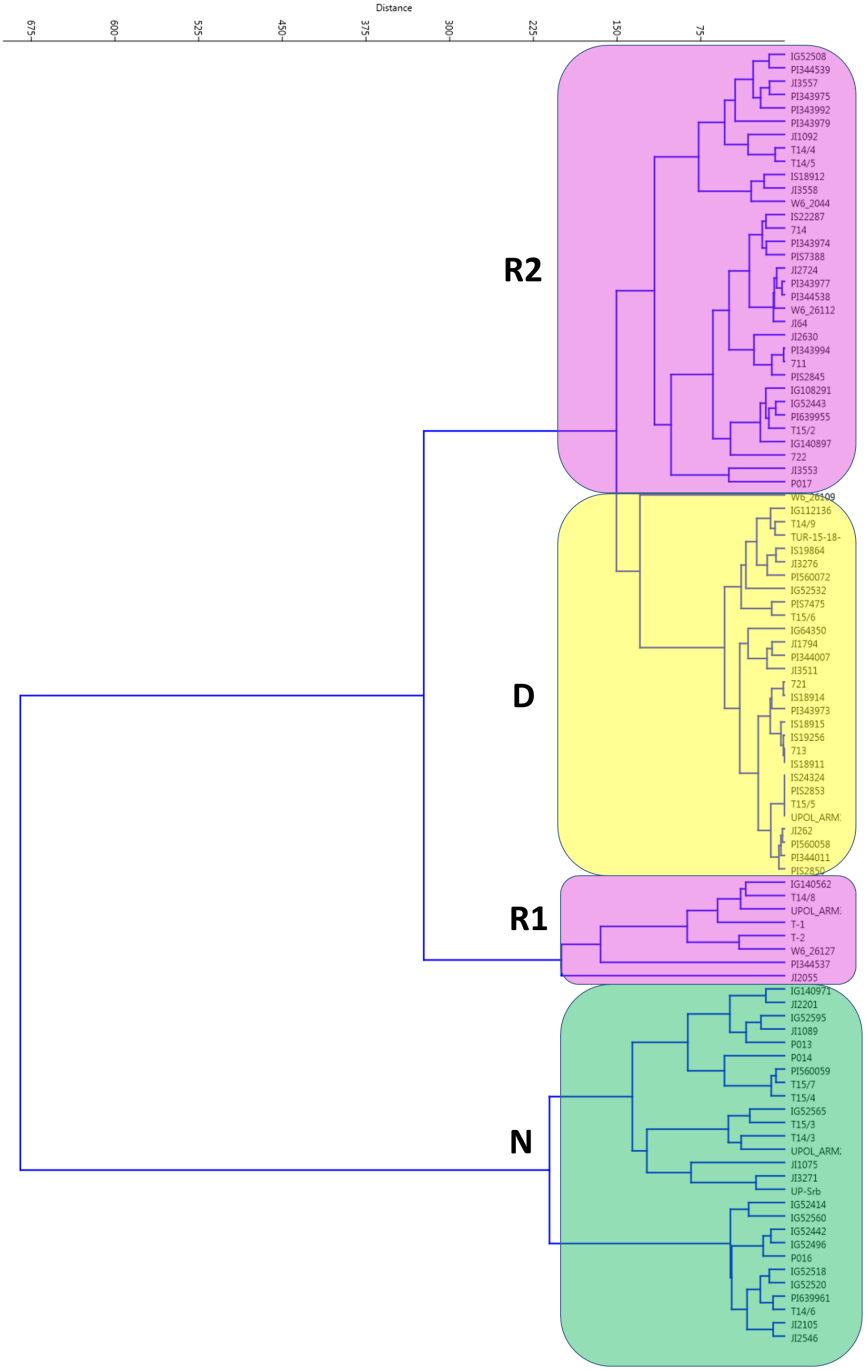
Cluster analysis of germination pattern of tested accessions in response to two alternate temperature regimes. Figure presents results of UPGMA of Euclidean distances of the area under the curves of calculated absolute germination distribution functions (AGDF) at 25/15 °C and 35/15 °C temperature regimes (See Table S2 for details). Clusters are coloured to visualise several categories of germination pattern (green: N, non-dormant, violet: R, responsive to temperature, red: D, dormant at both temperature regimes).

When germination responses at 25/15 °C and 35/15 °C were analyzed together, UPGMA clustering identified three groups (Fig. 2). One, with 28 non-dormant (N) accessions largely corresponding to N group at 25/15 °C treatment. The R1 group with eight accessions having large positive response at 35/15 °C (with change from 15% at 25/15 °C to up to 80% germination at 35/15 °C). Second responsive R2 group had 33 accessions with moderate response from 16% at 25/15° C to up to 60% germination at 35/15 °C). Finally, there were 29 accessions which remained dormant (e.g., with final germination bellows 20% at 35/15 °C, with 19 accession having less than 10%).

### Relationships between germination pattern, geography and environment

Each environmental variable was significantly spatially structured. Significant positive and negative spatial correlation coefficients were observed at small and large distance classes, respectively (Table S4).

**Figure 3:**
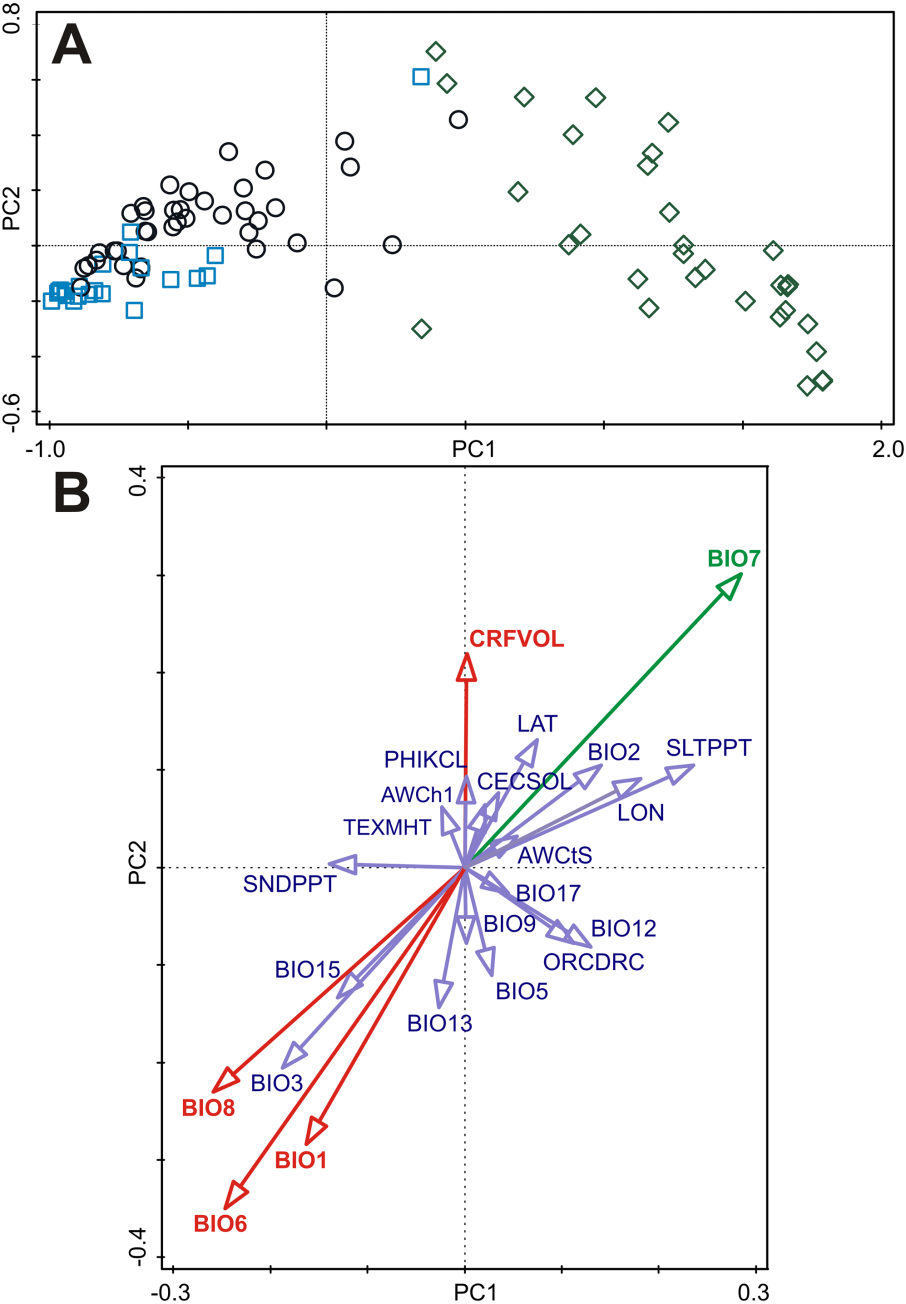
Principal component analysis (PCA) of the B-spline coefficients describing pattern of germination and multiple correlations of environmental variables with ordination axes. (A) The first and the second axes explain 87.3% and 5.6% of the total variation, respectively. Accessions are classified as either dormant (D, blue square), responsive (R, empty circle), or non-dormant (N, green diamond) according to the results of the cluster analyses (see Fig. 2). (B) Multiple correlations of environmental variables with the first and the second ordination axes of the PCA. Each arrow points in the direction of the steepest increase of the values for corresponding environmental variable. The angle between arrows indicates the sign of the correlation between the environmental variables. The length of the environmental variable arrows is the multiple correlation of that environmental variable with the ordination axes. Environmental variable in green is significantly correlated (spatial correlation) with both ordination axes. Environmental variables in red are significantly correlated (spatial correlation) with the second ordination axis. See Table S5 for Pearson correlation coefficients and corrected correlation coefficients (using Dutilleul method) of environmental variables with the first and the second ordination axes.

The first principal component of the PCA captured 87.3% of the variation of the B-spline coefficients matrix used to describe the germination pattern under both experimental temperature treatments and showed a clear gradient with dormant accessions on the left through responsive ones in the middle to the non-dormant accessions on the right of the ordination diagram (Fig. 3). Scores of accessions along the first ordination axis were therefore considered to be an indicator of the germination response of these accessions to experimental germination treatments (‘germination responsivity’). Germination responsivity was found to be significantly spatially structured. Significant positive spatial correlation coefficients were observed at two smallest distance classes (up to 100 and 200 km, respectively), negative spatial correlation was observed at the distance class 300–600 km, while nonsignificant spatial correlations were found for larger distance classes (Fig. S2).

Only subset of the environmental variables containing four bioclimatic variables (BIO1, BIO6, BIO7, BIO8) and one soil variable (CRFVOL) projected onto the two dimensional PCA diagram (PC1 and PC2 axes) were significantly correlated with at least one ordination axis after correction on spatial autocorrelation (Fig. 3, Table S5). However, only BIO7 was significantly correlated with PC1 axis representing germination responsivity (Fig. 3, Table S5).

**Figure 4:**
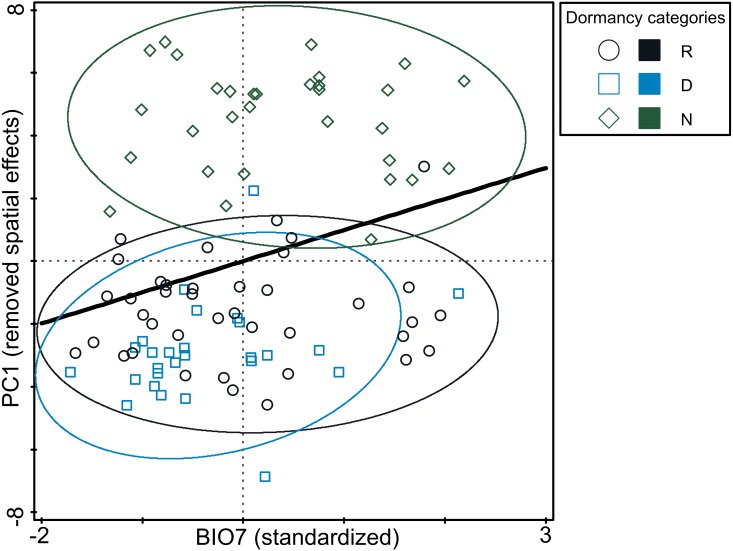
Relationship between germination responsivity of accessions in both temperature treatments and annual temperature range (BIO7). Germination responsivity is represented by residuals of scores along the first canonical ordination axis of RDA with one PCNM variable as an explanatory variable. Accessions are classified as either dormant (D; blue square), responsive (R; empty circle), or non-dormant (N; green diamond) according to the results of the cluster analyses (see Fig. 2). The ellipses were created based on a model of bivariate normal distribution of the dormancy class symbols (estimated from a variance-covariance matrix of their *X* and *Y* coordinates) to cover 95% of that distribution’ cases. Black line represent fitted Generalized Additive Model (response variable: PC1, predictor: BIO7, distribution: normal, link function: identity, fitted model deviance: 1,113.01 with 94.996 residual DFs, null model deviance: 1,207.34 with 96 residual *df*s, model AIC: 518.23, model test: *F* = 8.0, *P* = 0.0056).

The analysis of pure and shared effects of space (PCNM variables) and environmental variables (soil and climatic variables tested separately) on B-spline coefficient matrix representing germination pattern showed that all variables had an overall low explanatory power (space + soil variables: 13.6%; space + climatic variables: 17.4% of total variation in B-spline coefficient matrix; Table S6). PCNM variables accounted for more than a half of explained variation and always had significant effect on B-spline coefficients matrix. Shared effects between space and environmental variables (either soil or climatic) were non-significant on B-spline coefficients matrix. Overall pure effects of soil variables were non-significant on B-spline coefficients matrix, while climatic variables had significant overall pure effects on B-spline coefficients matrix. Using a forward selection procedure resulted in the stop of the selection process after the selection of BIO7 as the first and significant candidate term. Final RDA model thus included PCNM variables and BIO7 and explained 11.1% of total variation in B-spline coefficients matrix (Table S6). However, pure effect of BIO7 on B-spline coefficients matrix was weak (4.7% of total variation) and when three dormancy categories classified on the basis of cluster analysis were projected into resulting diagram, only poor separation among categories were visible (Fig. 4). Specifically, non-dormant and responsive accessions covered almost whole range of BIO7 while dormant accessions avoided occurring at sites with high values of BIO7, i.e., with larger temperature annual range.

**Figure 5:**
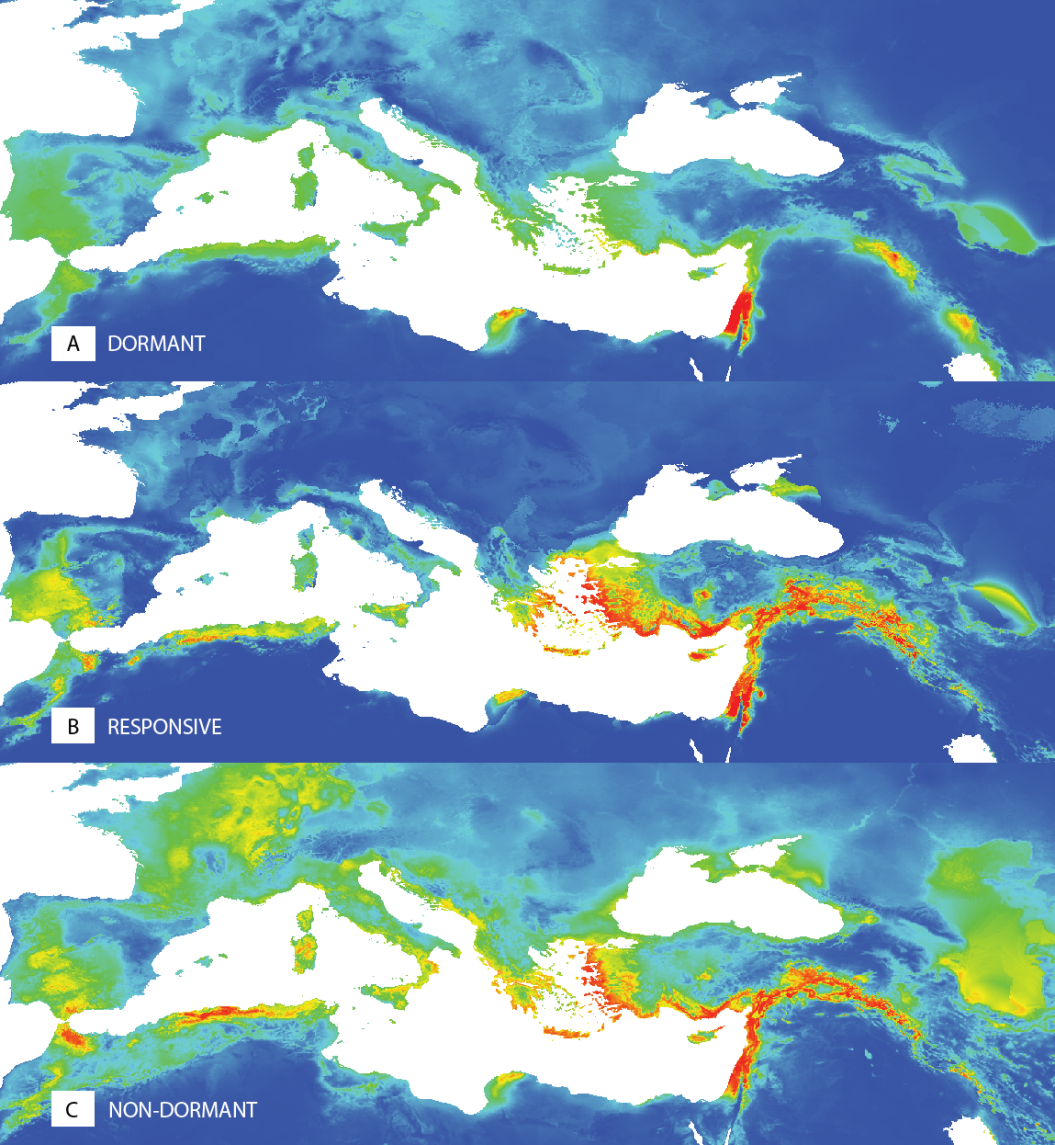
Predictions of niche models for the different dormancy groups. Colder colours represent areas of low probability of occurrence according to the models, while warmer colours correspond to areas of high probability, for dormant (A), responsive (B) and non-dormant (C) groups, respectively.

The ecological niche modelling of potential distributions of the dormant (D), temperature responsive (R) and the non-dormant (N) groups obtained at 35/15 °C testing showed the differences in spatial patterns (Fig. 5, Fig. S3). The dormant group (D) shows a more localized potential distribution. Results of the niche equivalence tests (Fig. S3) further suggest that any observed geographical differences among dormancy groups are not produced by an underlying divergence in the niche.

### Seed size and seed coat thickness relationship to dormancy level

**Figure 6:**
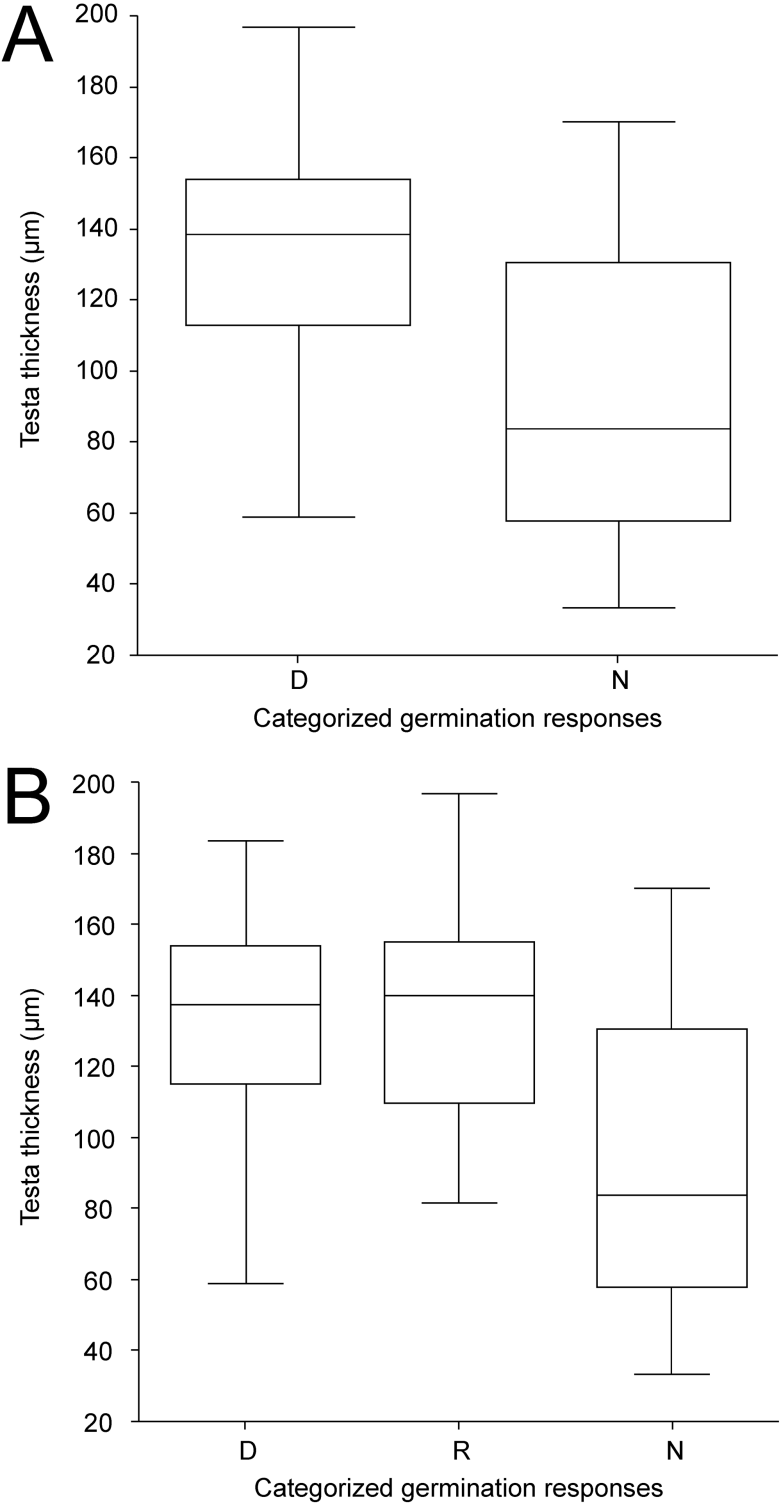
Box-plots of seed coat (testa) thickness of accessions classified into categories according to germination pattern under two temperature regimes. The panels compare dormant (D) and non-dormant (N) accessions tested under 25/15 °C temperature regime (A); and dormant (D), temperature responsive (R) and non-dormant (N) accessions under 35/15 °C temperature regime (B).

**Figure 7:**
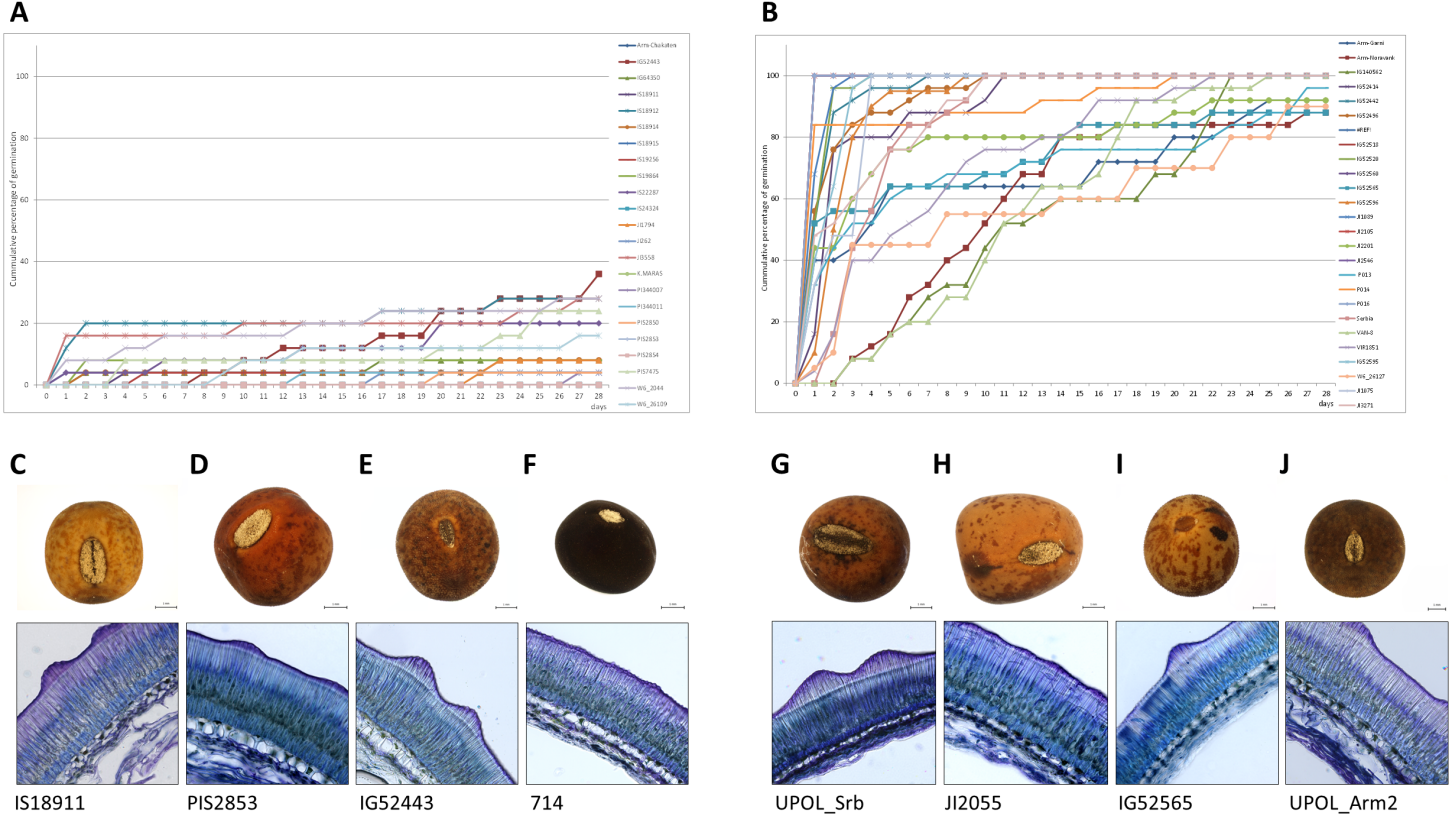
Comparison of dormant and non-dormant wild pea seed germination pattern, seed morphology and seed coat structure. The panels show the cumulative percentage over 28 days at 25/15 °C temperature regime of representative dormant (A) and non-dormant accessions (B) and seed micrographs and respective seed coat Toluidine stained sections at 40×magnification for dormant (C–F) and non-dormant (G–J) accessions.

Except for LWR having weak positive relationship towards non-dormancy, no clear relationships between categorized germination responses in the 25/15 °C treatment and seed morphological characters were observed (Fig. S4A). Similar results were obtained even when ordinal logistic regression with each morphological trait as covariate was employed (according to AIC simple logistic regression models were preferred over one multiple model); again the only significant covariate was LWR with *P* = 0.008. The ordinary logistic regression revealed a slight (positive) impact of the LWR covariate (*P* = 0.064) on dormancy release at 35/15 ° C treatment (Fig. S4B). Seed coat thickness differed between seed dormancy categories in the 25/15  °C treatment (Kruskal–Wallis test, *χ* ^2^ = 12.4, *P* < 0.001) with dormant accessions having thicker seed coat (median 139 µm, 95% CI [128–146] µm) than non-dormant ones (median 97 µm, 95% CI [65–120] µm) (Fig. 6A, Fig. 6B, Fig. 7, Figs. S5A, Figs. S5B). Similar results were obtained in the 35/15 °C treatment (Kruskal–Wallis test, *χ*^2^ = 11.7, *P* = 0.003), with both dormant (median 138 µm, 95% CI [123–150] µm) and responsive accessions (median 140 µm, 95% CI [110–152] µm) having similar and thicker seed coat than non-dormant ones (median 84 µm, 95% CI [58–117] µm). However, non-dormant accessions showed more variable seed coat thickness, reaching also the high values typical of dormant accessions (>80 µm), while seed coat thickness of dormant and responsive accessions did not fall below ca 80 µm (Figs. 6A, 6B, 7, Figs. S5A, S5B).

### Content of proanthocyanidins in the seed coat

The amount of soluble and insoluble PAs was measured in the isolated seed coat of mature dry seeds (Table S3). The total content varied from 1.21 to 4.70 mg g^−1^ of dry seed coat and showed weak tendency to be higher in the seed coat of dormant (mean ± SE; 2.18 ± 0.13  mg g^−1^) that those of non-dormant (mean ± SE; 1.77  ± 0.14 mg g^−1^) and responsive accessions (mean  ± SE; 1.87  ± 0.12 mg g^−1^; ANOVA, *F* = 2.64, *P* = 0.079). Soluble PA represented dominant component of total PA (94–99%) while insoluble PA were in minority (1–6%). However, ratio soluble/insoluble PA was not constant and increased with increasing total PA in all dormancy categories (Fig. S6). Correcting for total PA (covariate, *F* = 26.26, *P* < 0.001), dormancy categories recognized at 25/15 °C treatment significantly differed in sPA/inPA ratio (*F* = 4.6, *P* = 0.036) with dormant accessions having higher ratio (LS adjusted mean at mean total PA, 95% CI; 23.5, 20.8–26.4) than non-dormant ones (19.1, 16.3–22.2). Using the same model but with three dormancy categories (including responsive one; 35/15 °C treatment) showed similar trend to that of the previous model but the effect of dormancy category on sPA/inPA ratio was non-significant (total PA, *F* = 15.52, *P* < 0.001; dormancy categories, *F* = 2.59, *P* = 0.086) (Fig. S6). Custom comparison (D+R vs. N) showed that both dormant and responsive accessions have higher sPA/inPA ratio that non-dormant accessions (*F* = 4.46, *P* = 0.039).

## Discussion

We have tested the effect of temperature oscillation on wild pea seeds to mimic typical Mediterranean summer climate (Thompson, 2005) with often large diurnal temperature oscillations followed by main rainfall in the autumn season. Temperature and water availability are critical environmental factors regulating seed dormancy and germination (Probert, 2000; Vleeshouwers & Kropff, 2000). Temperature sensing determine the time of the year and depth of dormancy (Fenner & Thompson, 2005; Bewley et al., 2013; Baskin & Baskin, 2014; Batlla & Benech-Arnold, 2015; Renzi, Chantre & Cantamutto, 2018).

In the course of evaluation of germination data we have tested various commonly used coefficients (Ranal & Santana, 2006). It is known that there are difficulties in their interpretation and statistical analysis (Kader, 2005; Soltani et al., 2015). None of the commonly used measures, such as: cumulative percentage which is the static expression of germination behaviour at given time-point, LT50—a time to 50% of final germination (Soltani et al., 2015), coefficient of velocity—as a measure of the rate and time-spread of germination, mean germination time—a weighted mean of the germination time (Ranal & Santana, 2006) is able to differentiate between germination patterns (Table S2). Therefore in order to capture the complexity of germination we have used a *spline function* (function defined piecewise by polynomials) fitted to the data (Ramsay & Silverman, 2005; Van den Boogaart, Egozcue & Pawlowsky-Glahn, 2014). We have observed positive effect of higher temperature oscillation on release of wild pea seed dormancy (Fig. 2, Figs. S1A, S1B) similar to results obtained on some other legumes (*Lupinus*, *Trifolium* and *Medicago*) (Quinlivan, 1961; Quinlivan, 1966; Quinlivan, 1967; Quinlivan, 1968; Quinlivan & Millington, 1962) or *Phaseolus lunatus* (Degreef et al., 2002). However, detailed analyses of germination response to temperature treatments revealed marked differences in patterns of dormancy release among tested accessions. Intraspecific variation in germination patterns was little studied in legumes (reviewed in Baskin & Baskin, 2014); mostly only a few or even single accession was analysed. On the contrary, we have tested diverse wild pea accessions from the broad geographical area (Smýkal et al., 2017; Smýkal et al., 2018a; Trněný et al., 2018) originating from sites with markedly different climatic conditions. Such diverse environmental conditions might generate diverse selection pressures towards optimal germination strategies in specific environmental conditions (Rubiode Casas et al., 2017). Although the seed dormancy is highly heritable trait in pea (Hradilová et al., 2017; Cechová et al., 2017), the relationship between the environment and germination strategy is, however, largely unknown in pea.

Based on assessment of germination, we have obtained the range of germination response categories, from fully non-dormant (N) up to 100% germinating, through positively temperature responsive (R) to dormant (D) accessions (Fig. 2). Some of the non-dormant accessions could potentially be domesticated peas escaped from cultivation, and/or introgression between wild and domesticated pea as suggested by genetic diversity analysis (Trněný et al., 2018). They could however also be genuine wild peas without substantial seed dormancy, which occurs as part of natural variation, and which was likely used during early stages of pea domestication process (Trněný et al., 2018; Smýkal et al., 2018b). We can speculate about the alteration of seed coat composition, including the oxidation of fatty acids (Gamma-Arachchige et al., 2013; Cechová et al., 2017) or polyphenolic compounds (Hradilová et al., 2017) as well as physical changes (Janská et al., 2018) in the seed coat to occur during the temperature treatment.

### The relationship between environmental conditions and seed dormancy: patterns, potential limitations and alternative explanations

Despite expected relationships between pattern of seed dormancy release observed in our experiments and *in situ* environmental conditions of tested pea accessions, our results showed that germination responsivity (i.e., dormancy release) was weakly related to one climatic variable only: temperature annual range (BIO7). Specifically, dormant accessions does not occur at sites with large temperature annual range (Figs. 1, 3A, 3B, 4, Table S5). Using niche analysis we also showed that the dormant group (D) shows a localized potential distribution compared to the responsive (R) and non-dormant (N) groups (Fig. 5). This is a rather unexpected result, especially in the Mediterranean seasonal environment. This is further reinforced by the fact that the high probability areas of predicted occurrence constitute only a very small fraction of the high climate seasonality areas. Consequently, results of PCA, RDA and niche modeling in pea are not in agreement with metadata collected on wide range of legume species (Rubio de Casas et al., 2017) concerning the relationship between dormancy and seasonality. That study found that dormancy should increase in more seasonal environments. Of course, the niche patterns of both studies (Rubio de Casas et al., 2017; our study) could in part be attributed to scale differences (both spatial and taxonomic), but the need for an explanatory framework of wild pea dormancy in the Mediterranean region remains. Similar relationship between dormancy and environment as in pea was found by Wagmann et al. (2012) studying wild beet seed dormancy release. In contrary, two recent studies of Mediterranean wild lupines (Berger, Shrestha & Ludwig, 2017) and perennial woody legume (*Vachellia aroma*) along a precipitation gradient (Ferreras, Zeballos & Funes, 2017) showed no significant relationship between dormancy release and rainfall or temperature gradients. How could the dormancy-environment pattern we observed in pea be explained?

One set of explanations is related to (i) methodological constraints related to inaccurate estimations of *in situ* environmental conditions and (ii) specific conditions used in experimental germination treatments that might leave aside other important factors affecting *in situ* dormancy release in pea. Firstly, it can be speculated that the character of WorldClim data (average in term of time and also space) masks the micro-ecological pattern (Smýkal et al., 2018a). In geomorphologically complex regions, environmental conditions change considerably over short spatial scales, such that neighbouring populations can be subject to different selective pressures. This was found in the study of seed dormancy of Swedish *A. thaliana* accessions, with general relationship to the latitude and climate variables, with northern lines generally being less dormant than southern ones (Kerdaffrec & Nordborg, 2017) but with detected exceptions related possibly to local scale. Secondly, in their original habitat the plants grow in very distinct environment (temperature and rainfall patterns) and consequently the dormancy status of produced seeds might differ from seeds we have been testing in our common garden experiments, as shown in *Arabidopsis* (Edwards et al., 2017). This would require *on site* seed collection and/or reciprocal common garden experiment (Wagmann et al., 2012; Exposito-Alonso et al., 2018), both being essentially prevented by current plant genetic resources handling methodology (Nagoya protocol, Secretariat of the Convention on Biological Diversity United Nations Environmental Programme, 2011). Since we have not tested seeds in natural environment, we do not know if there is a cyclic pattern of germination as reported for several legumes with physical dormancy (Taylor, 1981; Taylor, 1996; Van Assche, Debucquoy & Rommens, 2003; Taylor, 2005). Seasonal testing of germination capacity of 14 annual legumes (of *Trifolium, Medicago, Melilotus, Vicia*) suggested the importance of chilling (low winter temperatures), prior to the submission to oscillating temperatures (Van Assche, Debucquoy & Rommens, 2003). Most of these taxa are temperate species, while in the case of Mediterranean and middle East origin wild pea *Pisum* sp*.* (Smýkal et al., 2017) chilling is expected to play only minor role as seedling establishment in natural conditions occurs from early to late autumn (recorded observations).

It also remains to be elucidated how a combination of temperature and moisture treatments influence the water entry, as shown for *Ipomoea lacunosa* (Jayasuriya et al., 2009) and comparison of wild and cultivated pea (Janská et al., 2018). The effect of substrate moisture was far less studied, partly because of problems with its control and manipulation. In natural conditions, the oscillations of temperature are accompanied by soil moisture changes. These are not fully recapitulated in laboratory experiments. Commonly used PEG solution (Michel & Kaufmann, 1973) provides only static conditions. In our experimental system we did not manipulate the water potential, and tested only water saturated conditions. Moreover, we used fully matured dry seeds from glasshouse grown plants to minimalize the seed source variability and to allow the experimental testing of seed responses and analysis of seed coat properties. In our study we used fully mature dry seeds obtained from glasshouse grown plants to minimalize the influence of the environment (Chen et al., 2014; Springthorpe & Penfield, 2015; Penfield & MacGregor, 2017; Renzi, Chantre & Cantamutto, 2018). Finally, it was found that both seed-maturation temperature and the timing of dispersal, strongly influenced germination behaviour (Edwards et al., 2017). If such situation is present in dehisced pea seeds under natural conditions remains to be tested. In addition to abiotic environmental (such as tested temperature or moisture) also biotic factors can have significant role. Thus we cannot exclude the action of microbes and fungus as recently demonstrated for *Lepidium didymium* (Brassicaceae) seeds released from dormancy by fungal degradation of the pericarp (Sperber et al., 2017). However, pea seed coats contain numerous antimicrobial substances including phenolic compounds (Pourcel et al., 2007; Hradilová et al., 2017; Raviv et al., 2017) which might prevent this.

Possible alternative explanation can be that pea seed dormancy is a bet-hedging mechanism in fluctuating (unpredictable) environments, in agreement with Volis & Bohrer (2013). Most of the high probability areas of the dormant (D) group are restricted to regions such as Israel, which has high summer temperatures and low temperature seasonality, a pattern also supported by the PCA and RDA results. These areas are very close to semi-desert environments and are characterized by higher unpredictability compared to the wet edge seasonal Mediterranean environment, which is more predictable. This is in agreement with models of Holst, Rasmussen & Bastiaans (2007) where seasonal fluctuations in temperature are considered the main regulatory factor of annual dormancy cycling (Bewley et al., 2013), while in semiarid and arid regions with high inter-annual rainfall variability, soil moisture is a major determinant for seedling emergence. As such, it could be concluded that for wild pea, dormancy is a bet-hedging mechanism developed to overcome the most unpredictable seasonal environments of the Mediterranean region. This explanation corresponds with the view that if conditions at germination are predictive of future survival and reproduction, then the value of bet-hedging decreases (Clauss & Venable, 2000). However, there is an area of overlap between the predicted distributions of the dormant (D), responsive (R) and the non-dormant (N) groups. It seems that this overlap is a mechanism of risk spreading, obtaining a long term selective advantage, which is a very successful strategy to adapt rapidly to changing environments. According to Philippi & Seger (1989), bet-hedging strategies are of two main forms: conservative and diversified. Conservative bet-hedging strategies tend to produce ever safer phenotypes, while diversifying bet-hedging strategies distribute the risk among two or more phenotypes. If seed dormancy is considered as a bet-hedging mechanism, then it can be viewed as the conservative form, producing the safest phenotypes; if we consider the niche overlap of the three groups, then the diversified, risk-spreading form is also apparent. Furthermore, the absence of strong environmental determination of environment-dormancy relationship, which is in consistency with the results of the niche equivalency tests, suggests that this bet-hedging mechanisms is functioning to cope with fine unpredictable fluctuations in similar environmental conditions.

The high probability areas of the non-dormant (N) group coincide with the Middle East and the Fertile Crescent, where the agricultural revolution and pea domestication took place (Trněný et al., 2018). Two hypotheses can be formulated with regard to this pattern. According to the first, mentioned above, the non-dormant group could be potentially domesticated pea escaped from cultivation, and/or an introgression group between wild and domesticated pea (Trněný et al., 2018). However, genomic analysis has revealed that only nine non-dormant accessions might be indeed genetically related to cultivated pea (IG52518, 52520, IG52442, IG52596, JI1089, JI2105, JI2201, JI2546 and P014) while the remaining 20 non-dormant accessions have clear genetic relationship to various clusters of wild *P. elatius* and are distant to cultivated pea (Trněný et al., 2018). On the other hand, if we assume that non-dormancy was a pre-domestication trait of wild pea even in very low frequencies within a bet-hedging diversified mechanism, our data support the view that during pea domestication such genotypes were selected and fixed in the pea crop.

### Seed size, seed coat thickness and proanthocyanidins content relationships to the dormancy

In our study we have found differences in total PA content and ratio of soluble to insoluble PAs in relationship to dormancy levels (Table S3, Fig. S6). Inhibitory effect of PAs in the seed coat on germination was found in *Sapium sebiferum* (Euphorbiaceae) where the seed coat extract or PAs significantly inhibited germination via alteration of gene expression (Afzal et al., 2017). This would be certainly the conclusion when non-pigmented cultivated pea varieties were compared to wild accessions (Hradilová et al., 2017). Moreover also in legumes, pigmented seeds imbibe slower than non-pigmented ones and also germinate later (Wyatt, 1977; Werker, Marbach & Mayer, 1979; Kantar, Pilbeam & Hebblethwaite, 1996). Notably, insoluble PAs were shown to contribute to *Rubus* seed dormancy (Wada, Kennedy & Reed, 2011). Variation in PA content in the cultivated pea seeds has been reported (Troszynska & Ciska, 2002) but not in comparison to wild peas. Our data of PA content (Fig. S5) are in agreement with early studies (Marbach & Mayer, 1974; Werker, Marbach & Mayer, 1979) reporting positive correlation in content of phenolics, activity of catechol oxidase and pea seed dormancy, although they compared only wild versus domesticated accessions. We can speculate about the alteration of seed coat composition, including the oxidation of fatty acids (Gamma-Arachchige et al., 2013; Cechová et al., 2017) or polyphenolic compounds (Hradilová et al., 2017) during the temperature treatment (Janská et al., 2018). Indeed in *Medicago truncatula* cells of the outer integument showed abundant accumulation of polyphenolic compounds which may impact seed permeability (Moïse et al., 2005). Our analysis was based on spectrophotometric measurements of total content and we cannot exclude that specific PAs can be more specifically associated with dormant seed coat, as revealed by detailed analysis of pea seed coat (Hradilová et al., 2017) as well as soybean (Zhou et al., 2010). It remains to be tested if there are any specific PA compounds related to dormancy levels in wild pea.

Intraspecific variation of seed size in relation to dormancy was so far only rarely tested. Comprehensive study of legume species (Rubio de Casas et al., 2017) showed that smaller, dormant seeds are favoured in the environments where the growing season is short, whereas in the tropics, large non-dormant seeds dominate. In our study, we have found no relationship between seed weight or seed size parameters and dormancy, except for length-to-width ratio (LWR) being slightly positively correlated with non-dormant seed types (Figs. S4A, S4B). There might be some influence of the shape of seeds (Fig. 7) but obviously not the size of seeds themselves. During crop domestication (Smýkal et al., 2018b) including the legumes (Berger, Shrestha & Ludwig, 2017), selection acted toward seed size increase. At intraspecific level, in the case of bird vetch (*Vicia cracca*) the positive relationship between seed size and germination (Eliášová & Münzbergová, 2014) was observed but in relation to the ploidy level. This is not a case of diploid *Pisum*. From a reproductive point of view, seed size trades off with seed number, small-seeded species clearly have the advantage in fecundity. The advantage of large seeds appears to be their tolerance of stresses such as shade or drought (Rees, 1996). If this operates in wild pea, it remains to be tested. Positive correlation between testa thickness and seed dormancy is in agreement with several reports (reviewed in Baskin & Baskin, 2014) and confirms our previous findings in pea (Hradilová et al., 2017; Janská et al., 2018).

## Conclusions

This study provides the framework for the study of environmental aspects of physical seed dormancy exploring also the observed germination pattern of seed coat structure and composition. New approach based on functional data analysis was successfully used and this can be recommended as a new analytical tool in seed dormancy/germination studies. In our experimental set up, pea seed germination responsivity in the *ex-situ* germination experiment (dormant → responsive → non-dormant) was weakly related with the increase of *in-situ* temperature annual range (BIO7). The significant differences between dormant and non-dormant seeds were found in the seed coat thickness and marginally also in the amount of proanthocyanidins in the seed coat, while the seed size was not related to the dormancy categories. Although it is established that temperature is the main driving force of dormancy release, a mechanistic and molecular mechanism are lacking. Dormant accessions are found in the environment with generally high annual temperature, smaller temperature variation, seasonality and milder winter. In general, dormant accessions are typical of narrower temperature profiles. Responsive accessions originate from the environment with lower annual temperature, colder winter and higher diurnal and seasonal temperature fluctuations. Ecological niche modelling showed a more localized potential distribution of dormant group and supports the view that the wild pea dormancy is part of a bet-hedging mechanism to cope with fine unpredictable environmental fluctuations in seasonal environments.

##  Supplemental Information

10.7717/peerj.6263/supp-1 Figure S1 Germination pattern of tested accessions Panels show UPGMA of Euclidean distances of B-spline coefficient germination matrix among studied accessions for 25/15 °C (A) and 35/15 °C (B) treatments.

10.7717/peerj.6263/supp-2 Figure S2 Spatial correlation analysis of germination responsivity of genotypes represented by the scores along the first ordination axis of PCA of B-spline coefficient Moran’s I spatial correlation statistics (±95% CI) is plotted against distance classes. Coefficients marked by black circle are significantly (*P* ≤ 0.05, Bonferroni correction) different from 0.

10.7717/peerj.6263/supp-3 Figure S3 Results of niche equivalency tests The grey bar histograms show the simulated values of niche overlap metrics D (left) and I (right), while the observed values for these metrics are represented by vertical red lines.

10.7717/peerj.6263/supp-4 Figure S4 Boxplots of morphological traits of seeds categorized according to their germination responses Panels show boxplots for categories of germination response tested at 25/15 °C (A) and 35/15 °C (B) temperature regimes. Explanations: Seeds area, AS (mm^2^), Perimeter length, PL (mm), Length, L (mm), Width, W (mm), Length-to-width ratio, LWR, Seed circularity, CS.

10.7717/peerj.6263/supp-5 Figure S5 Relationship between testa thickness and germination Plots show testa thickness in relation to germination categories (dormant, D, non-dormant N and responsive, R) tested at 25/15 °C (A) and 35/15 °C (B).

10.7717/peerj.6263/supp-6 Figure S6 The amount of total proanthocyanidins (PAs) and ratio of soluble to insoluble PAs in the seed coat Comparison of theamount of PAs in-dormant (D), non-dormant (N) and temperature responsive (R) accessions.

10.7717/peerj.6263/supp-7 Table S1 GPS coordinates and extracted WorldClim and SoilGrid values for 97 studied wild pea accessions

10.7717/peerj.6263/supp-8 Table S2 Germination coefficients and calculated B-spline coefficients characterising absolute germination distribution function (AGDF) for each accession under two temperature treatments

10.7717/peerj.6263/supp-9 Table S3 Amount of soluble, insoluble and total proanthocyanidins, seed coat thickness and hundred seeds weight HSW (g)

10.7717/peerj.6263/supp-10 Table S4 Spatial correlation analysis of each environmental variable (see Table S1) Moran’s I spatial correlation statistics (±SD) is for each distance class is reported. Coefficients in bold are significantly (*P* ≤ 0.05, Bonferroni correction) different from 0.

10.7717/peerj.6263/supp-11 Table S5 Correlations and spatial correlations (using modified version of the *t*-test according to Dutilleul et al. (1993) of each environmental variable with the first two ordination axes of the PCA of the B-spline coefficients matrix (Fig. 3) Scores of accessions along the first ordination axis were considered to be an indicator of the germination response of these accessions to experimental germination treatments (‘germination responsivity’).

10.7717/peerj.6263/supp-12 Table S6 Pure and shared effects of environmental (soil and climatic variables tested separately) and space (PCNM variable) and of all variables combined, on the matrix of B-spline coefficients, characterising germination pattern of pea accessions in two experimen Degrees of freedom (*df*), test statistics (F), P value and adjusted coefficient of determination (*R*^2^) values are given for each variable. The significances were tested using a Monte Carlo permutation test with 999 permutations.

## Additional Information and Declarations

### Data Availability

The following information was supplied regarding data availability: The raw measurements are provided in Tables S1–S6.
