# Supplementary figures and images for: Variation in wild pea (Pisum sativum subsp. elatius) seed dormancy and its relationship to the environment and seed coat traits
Source: PeerJ. 2019 Jan 14;7:e6263. doi: 10.7717/peerj.6263 (PMC6336014; doi:10.7717/peerj.6263)

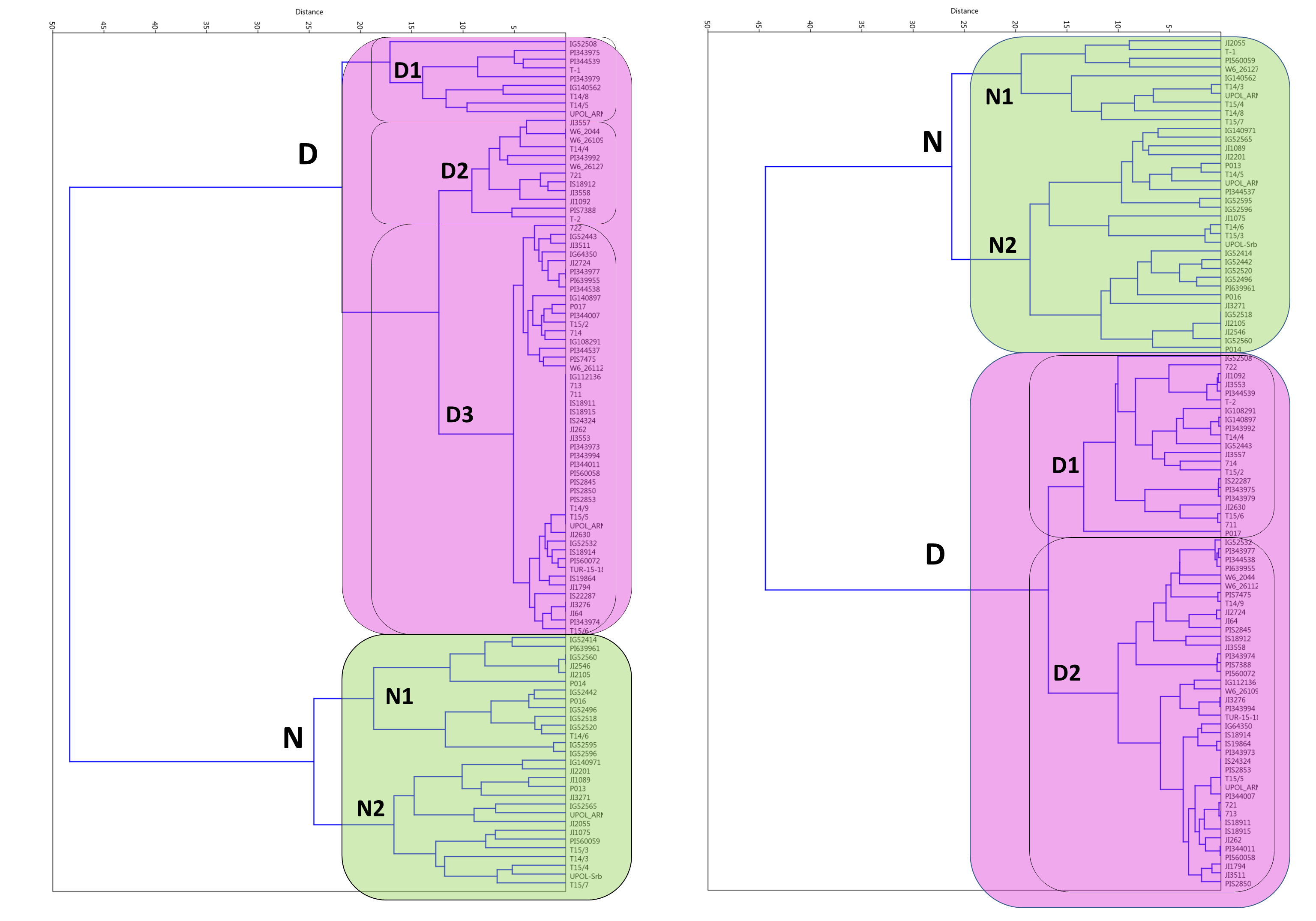

Supplement: Figure S1 — Panels show UPGMA of Euclidean distances of B-spline coefficient germination matrix among studied accessions for 25/15 °C (A) and 35/15 °C (B) treatments. [file peerj-07-6263-s001.png]

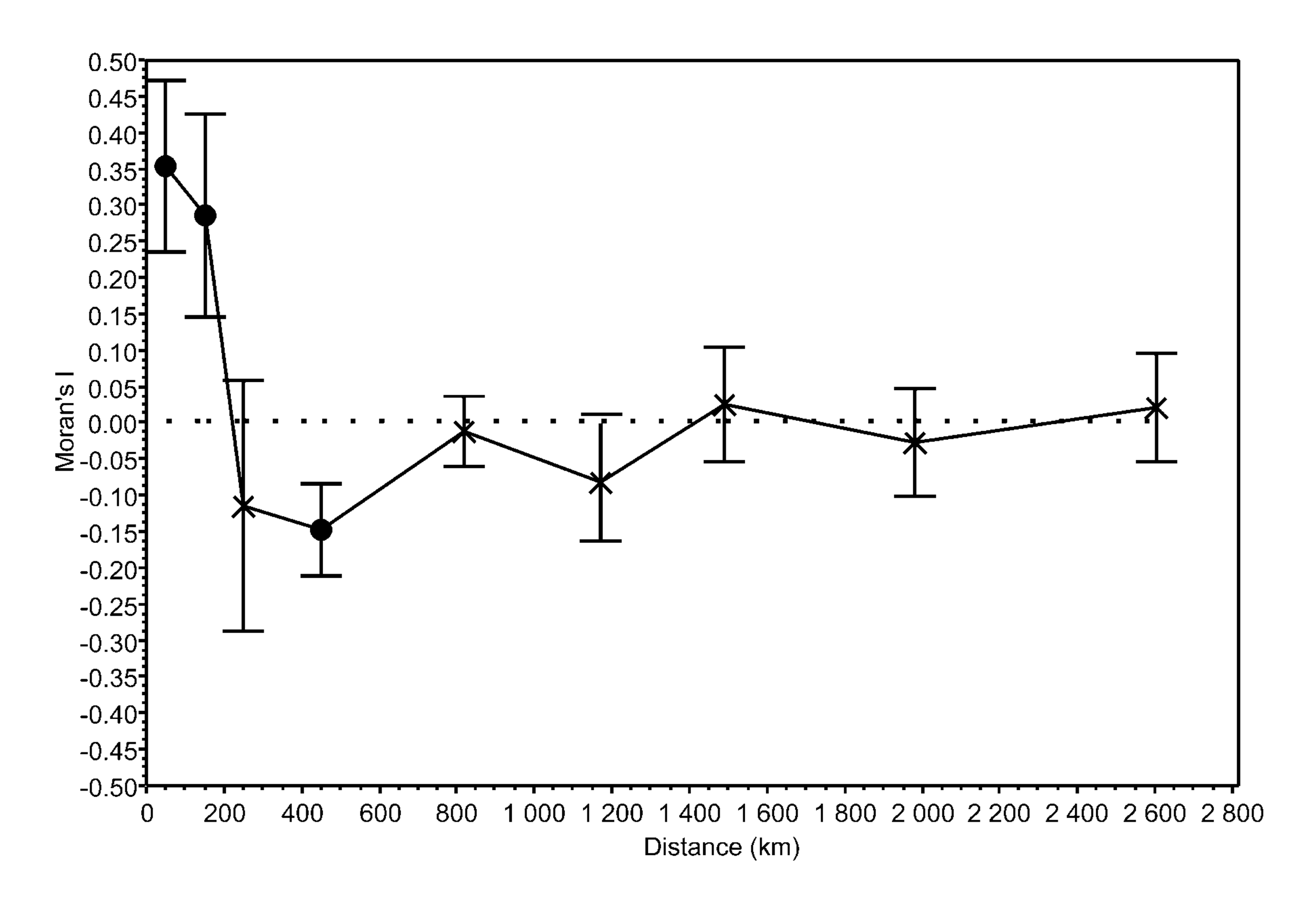

Supplement: Figure S2 — Moran’s I spatial correlation statistics (±95% CI) is plotted against distance classes. Coefficients marked by black circle are significantly (P ≤ 0.05, Bonferroni correction) different from 0. [file peerj-07-6263-s002.png]

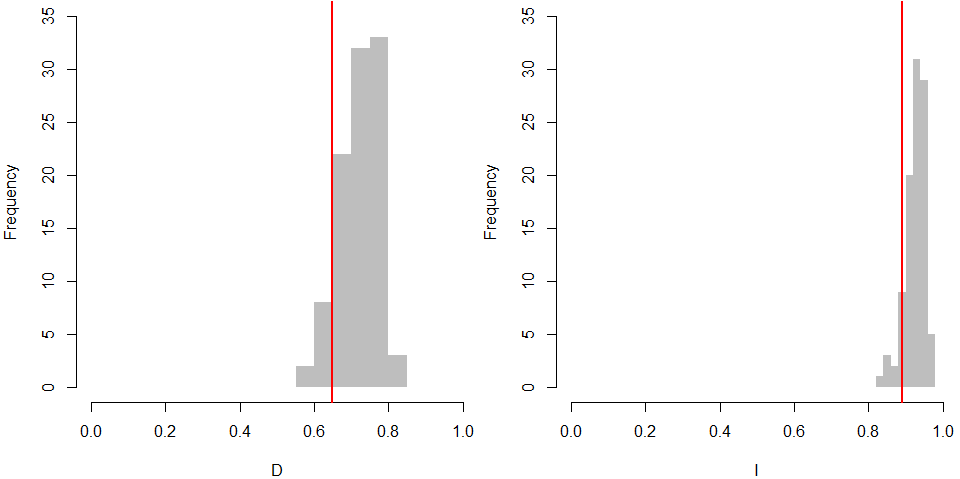

Supplement: Figure S3 — The grey bar histograms show the simulated values of niche overlap metrics D (left) and I (right), while the observed values for these metrics are represented by vertical red lines. [file peerj-07-6263-s003.png]

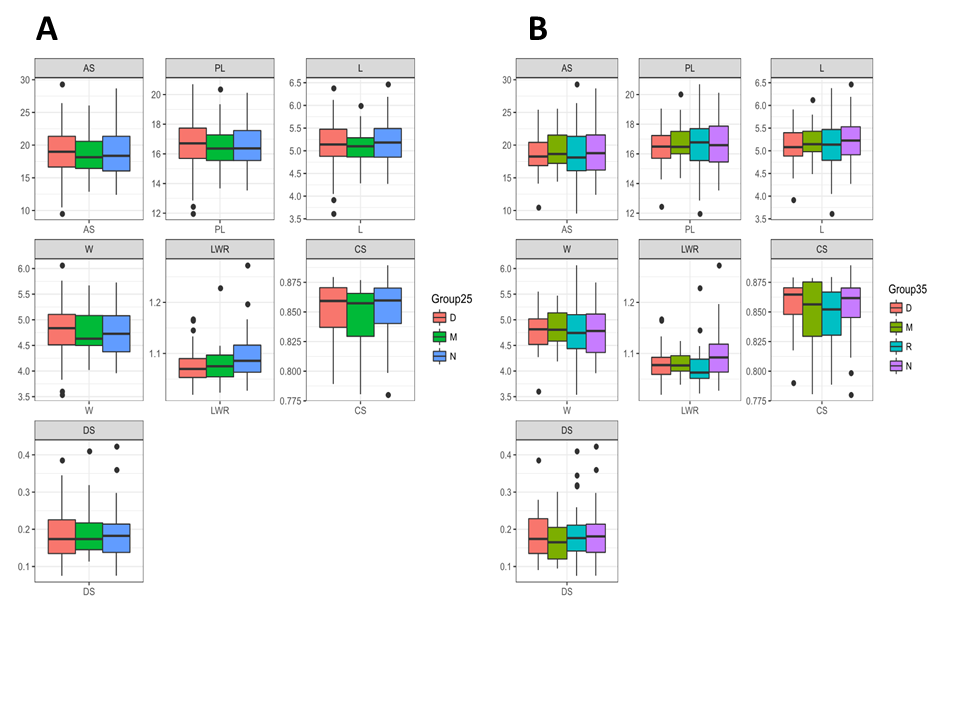

Supplement: Figure S4 — Panels show boxplots for categories of germination response tested at 25/15 °C (A) and 35/15 °C (B) temperature regimes. Explanations: Seeds area, AS (mm2), Perimeter length, PL (mm), Length, L (mm), Width, W (mm), Length-to-width ratio, LWR, Seed circularity, CS. [file peerj-07-6263-s004.png]

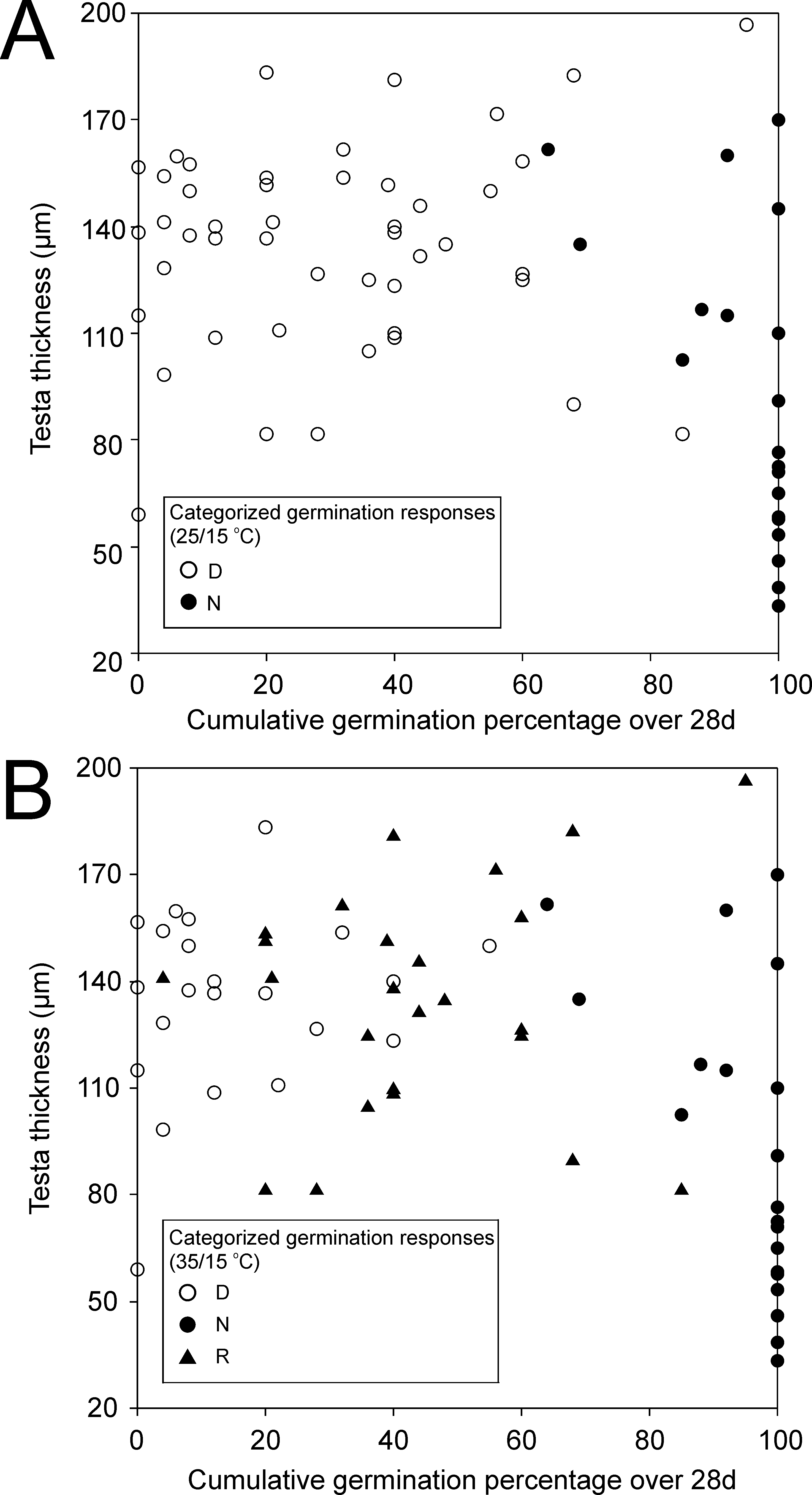

Supplement: Figure S5 — Plots show testa thickness in relation to germination categories (dormant, D, non-dormant N and responsive, R) tested at 25/15 °C (A) and 35/15 °C (B). [file peerj-07-6263-s005.png]

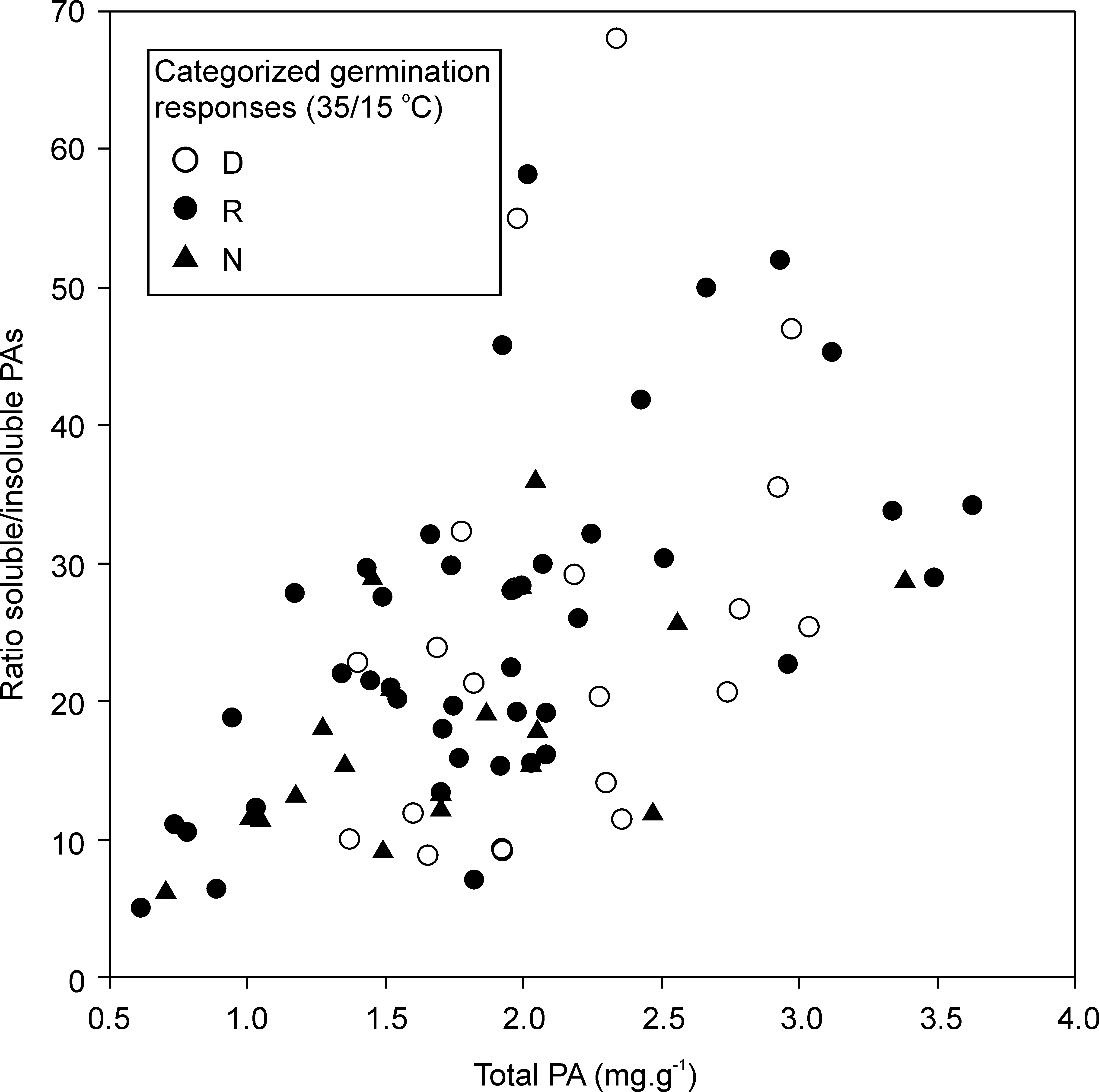

Supplement: Figure S6 — Comparison of theamount of PAs in-dormant (D), non-dormant (N) and temperature responsive (R) accessions. [file peerj-07-6263-s006.png]
